# Exploring Symptom Domains of Postoperative Bowel Dysfunction by Integrating LARS and Wexner Scores: A Longitudinal Factor‐Analytic Study

**DOI:** 10.1002/ags3.70289

**Published:** 2026-09-28

**Authors:** Yoshiko Matsumoto, Issei Takeshita, Keiichi Shiokawa, Kurumi Sahara, Taro Munechika, Ken Nagata, Hideki Nagano, Hiroyuki Takahashi, Suguru Hasegawa

**Affiliations:** ^1^ Department of Gastroenterological Surgery Fukuoka University Hospital Fukuoka Japan

**Keywords:** bowel dysfunction, factor analysis, fecal incontinence, LARS, rectal cancer

## Abstract

**Background:**

Postoperative bowel dysfunction after sphincter‐preserving surgery for rectal cancer is heterogeneous and may not be fully captured by composite scores such as the low anterior resection syndrome (LARS) and Wexner scores. This study aimed to identify clinically relevant symptom domains and examine their associated clinical factors.

**Methods:**

We analyzed 320 patients who underwent low anterior resection with longitudinal bowel function assessment. Correlations among individual LARS and Wexner components were evaluated using Spearman's correlation and hierarchical clustering. Exploratory factor analysis using pooled postoperative observations was performed to identify latent symptom domains, and multivariable mixed‐effects models were used to examine associated clinical factors.

**Results:**

Correlation analysis demonstrated heterogeneous inter‐item relationships, and factor analysis identified three domains: incontinence, urgency/clustering, and gas control. In multivariable analysis, greater incontinence severity was associated with age 50–70 years, BMI ≥ 23, neoadjuvant chemotherapy, radiotherapy, very low anterior resection, and a transanal approach. Greater urgency/clustering severity was associated with radiotherapy, whereas age ≥ 70 years was associated with lower severity. Gas‐related symptoms may also represent a partially distinct domain. Postoperative time was a significant determinant of all three domains, with symptom severity remaining higher at 6, 12, and 24 months than preoperatively.

**Conclusions:**

Postoperative bowel dysfunction, as captured by the LARS and Wexner scores, appears to consist of multiple symptom domains with different clinical determinants. Patients with similar composite scores may have different symptom profiles requiring different management strategies. A domain‐based approach may support individualized functional assessment and treatment after rectal surgery.

## Graphical abstract

Item‐level factor analysis of the LARS and Wexner scores in 320 patients undergoing sphincter‐preserving rectal resection identified three distinct symptom domains: incontinence, urgency/clustering, and gas control. These domains had different clinical determinants: radiotherapy was associated with greater severity of both incontinence and urgency/clustering, whereas very low anterior resection and a transanal approach were associated with greater incontinence. Age showed contrasting domain‐specific associations. Patients with similar composite scores may therefore have different symptom profiles, supporting a domain‐based approach to individualized functional assessment after rectal surgery.

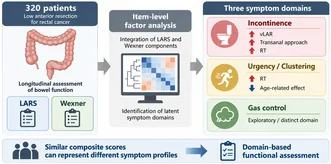

## 1 Introduction

With the widespread adoption of sphincter‐preserving surgery for rectal cancer, postoperative bowel dysfunction has become a major determinant of patient quality of life. Low anterior resection syndrome (LARS) comprises a spectrum of symptoms, including fecal incontinence, increased stool frequency, urgency, and clustering, which may persist long‐term and impair daily activities [1].

The LARS score, a patient‐reported outcome measure consisting of five symptom items, is widely used to assess symptom severity and has been validated in Japanese populations [2, 3]. In parallel, the Cleveland Clinic Florida Fecal Incontinence Score (Wexner score) is commonly used to evaluate incontinence [4]. However, because the Wexner score primarily reflects incontinence‐related symptoms, it may not fully capture evacuation‐related features such as urgency and clustering that are characteristic of LARS. Thus, combining these scores may provide complementary insights into postoperative bowel dysfunction by reflecting distinct symptom domains.

Several clinical and treatment‐related factors, including preoperative radiotherapy, low tumor location, and anastomotic height, have been consistently identified as major risk factors for LARS [5, 6]. These findings support the multifactorial nature of postoperative bowel dysfunction, involving structural, neurological, and functional components. In contrast, the influence of patient‐related factors such as age remains less clearly defined, with inconsistent findings across studies [5, 7].

Importantly, reliance on composite scores alone may obscure heterogeneity in symptom patterns. Even among patients with similar overall scores, the dominant symptom profile may differ, suggesting that LARS and Wexner components can be organized into distinct domains, such as incontinence‐related and evacuation‐related symptoms.

Therefore, the aim of this study was to identify clusters of symptom components with similar patterns across the LARS and Wexner scores using factor analysis, and to examine the clinical determinants associated with each derived symptom domain.

## 2 Methods

This study was a retrospective analysis of a prospectively maintained single‐center database. We included 320 patients who underwent low anterior resection for rectal tumors at Fukuoka University Hospital between 2016 and 2025 and had available LARS and Wexner score data at two or more assessment points, including the preoperative and/or postoperative time points. In patients with a diverting stoma, postoperative time points were defined relative to stoma closure. Exclusion criteria were high anterior resection, abdominoperineal resection, Hartmann's procedure, combined resection of other organs, and cases in which the diverting stoma had not been closed. Procedures were classified according to the anatomical level of the anastomosis. Very low anterior resection (vLAR) was defined as a resection in which the hiatal ligament was divided, dissection was carried into the anal canal, and a stapled anastomosis was created within the anal canal itself; low anterior resection (LAR) was defined as a resection in which the anastomosis was located above the anal canal. All anastomoses in this cohort were created using a stapling technique; intersphincteric resection and hand‐sewn coloanal anastomosis were not included. This classification was assigned by the operating surgeon at the time of surgery and recorded prospectively in the database.

The following variables were extracted from the prospectively collected database: age, sex, body mass index (BMI), height, weight, American Society of Anesthesiologists physical status (ASA‐PS), Eastern Cooperative Oncology Group performance status (ECOG‐PS), disease type, clinical stage, preoperative therapy, endoscopic passage, surgical approach, procedure type, use of a transanal approach, occurrence of anastomotic leakage, presence of diverting stoma, and its indication.

### 2.1 Statistical Analysis

Pairwise correlations among Wexner and LARS components were assessed using Spearman's rank correlation coefficients (*ρ*) to evaluate inter‐item relationships and identify patterns of association between symptom components.

Exploratory factor analysis was performed to identify latent domains underlying postoperative bowel dysfunction. Because the original LARS score uses non‐linear item weights designed to optimize total‐score classification rather than to evaluate structural relationships among individual items, each LARS component was recoded into an ordinal severity scale preserving the rank order of the original response categories (Table S1). This step was intended to reduce disproportionate weighting of selected items and to facilitate item‐level analysis of latent symptom structure. To characterize the overall latent structure of postoperative bowel dysfunction, the factor analysis was performed using pooled postoperative observations obtained at 6, 12, and 24 months after surgery. Factor extraction was performed using the maximum likelihood method, and the number of factors was determined based on eigenvalues > 1.0 and inspection of the scree plot. Quartimin rotation was applied to permit correlations among factors, and variables with factor loadings ≥ 0.30 were considered meaningful contributors. Factor scores were estimated using the regression method and treated as standardized latent continuous variables representing domain‐specific symptom severity. Scores were oriented such that higher values indicated greater severity in each domain (Factor 1: incontinence; Factor 2: urgency/clustering; Factor 3: gas control).

Multivariable mixed‐effects models were constructed to identify clinical and temporal determinants of each domain‐specific symptom severity. Patient ID was included as a random effect to account for repeated measures, and fixed effects included demographic, clinical, treatment‐related variables, and postoperative time (preoperative, 6, 12, and 24 months). Regression coefficients (*β*) indicate changes in domain‐specific severity, with positive values reflecting worsening symptoms. Questionnaire response rates at each assessment point are shown in Table S2. All available observations were included in the models without imputation; the mixed‐effects framework accommodates unbalanced longitudinal data under a missing‐at‐random assumption. No formal analysis of attrition was performed.

All statistical analyses were performed using JMP software (SAS Institute Inc., Cary, NC, USA). This study was conducted in accordance with the Declaration of Helsinki and approved by the Institutional Review Board of Fukuoka University Hospital (approval number: 16‐5‐04). Informed consent was obtained from all participants.

## 3 Results

### 3.1 Patient Characteristics

Among the 320 patients (Table 1), the mean age was 64.0 ± 11.3 years, and 194 (60.6%) were male. The mean BMI was 22.6 ± 3.6 kg/m^2^. Clinical Stage III–IV disease was observed in 52.5%. Preoperative therapy was administered in 14.4% of patients, including radiotherapy in 6.3%. Regarding surgical characteristics, robotic surgery was performed in 79.4%, very low anterior resection (vLAR) in 20.9%, and a transanal approach was used in 2.8%. A diverting stoma was created in 32.5% of patients and anastomotic leakage occurred in 6.3%. Questionnaire response rates at each assessment point are shown in Table S2.

**TABLE 1:** Patient characteristics (*N* = 320).

Variable	Category	Value
Age (years)		64.0 ± 11.3
	Median (IQR)	65.6 (55.3–71.8)
Sex	Male	194 (60.6%)
	Female	126 (39.4%)
BMI (kg/m^2^)		22.6 ± 3.6
ASA‐PS	1	190 (59.4%)
	≥ 2	130 (40.6%)
ECOG‐PS	0	266 (83.4%)
	≥ 1	53 (16.6%)
Disease type	Adenocarcinoma	302 (94.4%)
	NET/NEC/Schwannoma	18 (5.6%)
Clinical stage	≤ Stage II	152 (47.5%)
	≥ Stage III	168 (52.5%)
Preoperative therapy	None	274 (85.6%)
	NAC	26 (8.1%)
	Radiotherapy	20 (6.3%)
Endoscopic passage	Passable	297 (92.8%)
	Difficult	23 (7.2%)
Surgical approach	Robotic	254 (79.4%)
	Laparoscopic	66 (20.6%)
Transanal approach	None	311 (97.2%)
	Ta	9 (2.8%)
Procedure	LAR	253 (79.1%)
	vLAR	67 (20.9%)
Diverting stoma (DS)	DS−	216 (67.5%)
	DS+	104 (32.5%)
Stoma indication	Intraoperative DS	67 (64.4%)
	Preoperative (decompression)	25 (24.0%)
	Postoperative complication	12 (11.5%)
Anastomotic leakage	None	300 (93.8%)
	Grade 2	7 (2.2%)
	Grade 3a	2 (0.6%)
	Grade 3b	11 (3.4%)

*Note:* Data are presented as mean ± standard deviation or number (%).

Abbreviations: ASA‐PS, American Society of Anesthesiologists physical status; BMI, body mass index; DS, diverting stoma; ECOG‐PS, Eastern Cooperative Oncology Group performance status; NAC, neoadjuvant chemotherapy.

### 3.2 Correlation Structure of Wexner and LARS Components

Pairwise correlations among the components of the Wexner and LARS scores are presented in Table 2 and visualized as a clustered heatmap in Figure 1.

**TABLE 2:** Pairwise Spearman correlation matrix among individual components of the Wexner and LARS scores.

	Variable	Wexner score					LARS score				
		Solid incont.	Liquid incont.	Gas incont.	Pad use	Lifestyle alter.	Flatus incont.	Liquid incont.	Frequency	Clustering	Urgency
Wexner score	Solid incontinence	—									
	Liquid incontinence	0.36	—								
	Gas incontinence	0.34	0.37	—							
	Pad use	0.4	0.47	0.33	—						
	Lifestyle alteration	0.32	0.42	0.34	0.49	—					
LARS score	Gas incontinence	0.2	0.24	**0.58**	0.26	0.3	—				
	Liquid incontinence	0.29	**0.71**	0.28	0.37	0.38	0.25	—			
	Frequency	0.13	0.16	0.12	0.16	0.21	0.16	0.12	—		
	Clustering	0.13	0.25	0.25	0.2	0.23	0.27	0.27	0.18	—	
	Urgency	0.18	0.36	0.28	0.26	0.32	0.35	0.35	0.22	**0.59**	—

*Note:* Values represent Spearman's rank correlation coefficients (*ρ*). Bold values indicate strong correlations (*ρ* ≥ 0.5).

**FIGURE 1:**
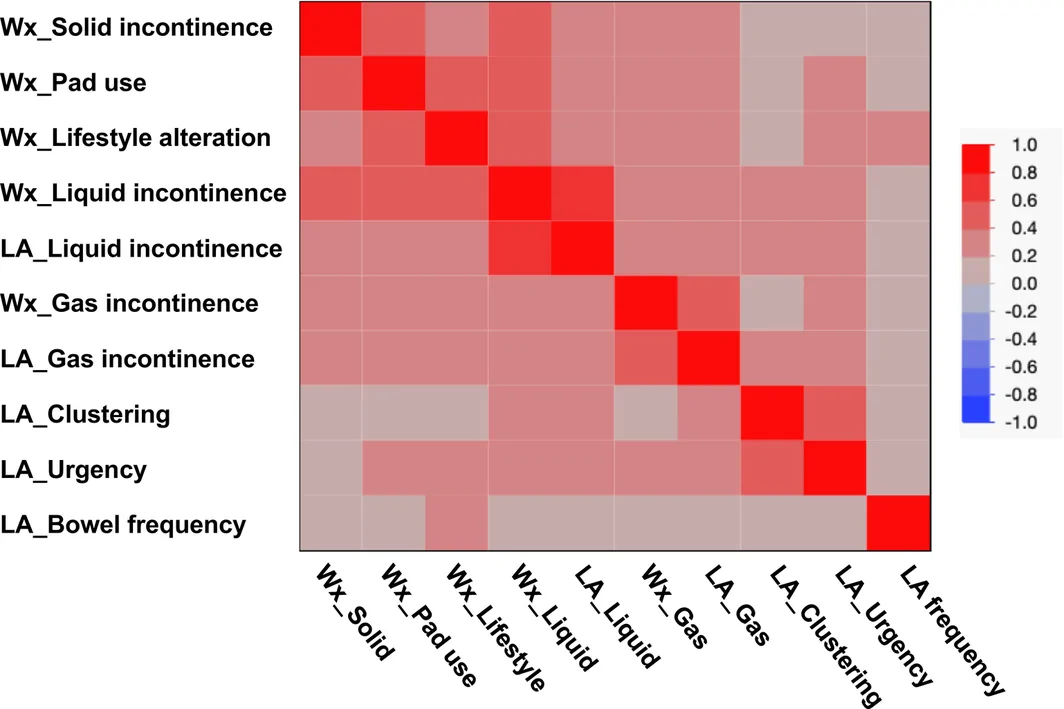
Clustered correlation matrix of Wexner and LARS components. Pairwise correlations among Wexner (Wx) and LARS (LA) components are shown as a heatmap based on Spearman's correlation coefficients. Variables were reordered using hierarchical clustering of the correlation matrix (Table 2) to highlight groups of related symptoms. Color intensity reflects the strength of correlation.

The correlation matrix demonstrated a heterogeneous pattern of associations. Strong correlations were observed between Wexner liquid incontinence and LARS liquid stool incontinence (*ρ* = 0.71), between Wexner gas incontinence and LARS flatus incontinence (*ρ* = 0.58), and between urgency and clustering within the LARS score (*ρ* = 0.59). In contrast, some components, particularly frequency, showed relatively weak correlations with other variables, suggesting a more diffuse relationship across symptom domains.

Reordering of variables using hierarchical clustering in Figure 1 revealed grouping of related components, indicating that symptoms tend to aggregate into several clusters with similar correlation patterns. These findings suggest the presence of partially overlapping but distinct domains of postoperative bowel dysfunction and provide the rationale for subsequent factor analysis.

### 3.3 Factor Analysis

The overall Kaiser–Meyer–Olkin (KMO) measure was 0.782, indicating acceptable sampling adequacy for factor analysis. Bartlett's test of sphericity was significant (*χ* ^2^ = 2352.437, df = 45, *p* < 0.001), confirming that the correlation structure was suitable for factor analysis. Based on the eigenvalues and scree plot, a three‐factor solution was retained and analyzed using the maximum likelihood method with Quartimin rotation. Although the three‐factor structure was considered clinically interpretable, the model fit indices suggested that it should be regarded as exploratory.

After rotation, Factor 1 showed high loadings for incontinence‐related items, including Wexner liquid incontinence, LARS liquid stool incontinence, pad use, solid stool incontinence, and lifestyle alteration, and was interpreted as an incontinence domain. Factor 2 was characterized primarily by high loadings for LARS urgency and clustering, with a smaller contribution from bowel frequency, and was interpreted as an urgency/clustering domain. Factor 3 showed high loadings for Wexner gas incontinence and LARS flatus incontinence and was interpreted as a gas control domain (Figure 2). Detailed rotated factor loadings are provided in Table S3.

**FIGURE 2:**
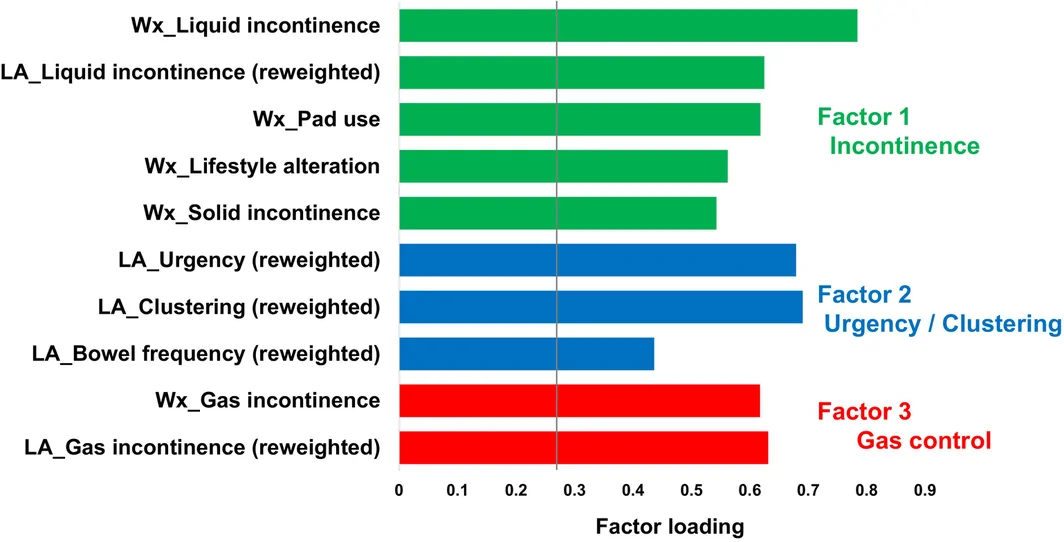
Factor structure of Wexner and LARS components. Factor loadings derived from exploratory factor analysis of Wexner (Wx) and LARS (LA) components are shown. Three factors were identified, representing incontinence, urgency/clustering, and gas control symptoms. Higher loadings indicate stronger contributions to each factor. LARS components were reweighted according to symptom severity prior to analysis.

These three factors accounted for 64.7% of the total variance (Factor 1: 27.6%, Factor 2: 18.8%, Factor 3: 18.4%). Higher factor scores were oriented to indicate greater symptom severity within each domain.

### 3.4 Determinants of the Three Symptom Domains of Postoperative Bowel Dysfunction

The results of the multivariable mixed‐effects analysis are summarized in Table 3, and the corresponding forest plots are provided in Figure S1. For Factor 1 (incontinence), greater severity was independently associated with age 50–70 years versus < 50 years (*β* = 0.292, 95% CI 0.069–0.515, *p* = 0.010), BMI ≥ 23 (*β* = 0.167, 95% CI 0.013–0.322, *p* = 0.034), preoperative chemotherapy (NAC vs. none: *β* = 0.360, 95% CI 0.033–0.688, *p* = 0.031), radiotherapy (RT vs. none: *β* = 0.503, 95% CI 0.171–0.834, *p* = 0.003), very low anterior resection (vLAR vs. LAR: *β* = 0.437, 95% CI 0.236–0.638, *p* < 0.001), and a transanal approach (Ta vs. none: *β* = 0.860, 95% CI 0.300–1.420, *p* = 0.003).

**TABLE 3:** Differential determinants of the three symptom domains of postoperative bowel dysfunction: a multivariable mixed‐effects analysis.

Variable	Category	Factor 1 incontinence *β* (95% CI)	*p*	Factor 2 (Clustering/urgency) *β* (95% CI)	*p*	Factor 3 (Gas control) *β* (95% CI)	*p*
Sex	Female vs. Male	0.140 (−0.022, 0.303)	0.0903	−0.057 (−0.215, 0.100)	0.4719	0.200 (0.039, 0.360)	0.0148
Age	50–70 vs. < 50	0.292 (0.069, 0.515)	0.0104	0.074 (−0.149, 0.296)	0.5157	0.043 (−0.174, 0.259)	0.6989
	≥ 70 vs. < 50	0.232 (−0.009, 0.473)	0.0594	−0.257 (−0.495, −0.020)	0.0339	−0.036 (−0.271, 0.198)	0.7612
ECOG‐PS	≥ 1 vs. 0	−0.142 (−0.364, 0.079)	0.2065	−0.196 (−0.411, 0.018)	0.0725	0.089 (−0.129, 0.307)	0.4208
ASA‐PS	≥ 2 vs. 1	0.039 (−0.126, 0.204)	0.6433	−0.056 (−0.215, 0.104)	0.4931	0.110 (−0.053, 0.273)	0.1837
BMI	≥ 23 vs. < 23	0.167 (0.013, 0.322)	0.034	0.109 (−0.040, 0.258)	0.1509	0.037 (−0.115, 0.189)	0.632
Clinical stage	≥ Stage III vs. ≤ Stage II	0.067 (−0.092, 0.225)	0.4108	0.045 (−0.109, 0.198)	0.5653	−0.010 (−0.167, 0.147)	0.9006
Endoscopic passage	Difficult vs. normal	0.072 (−0.233, 0.377)	0.6424	−0.024 (−0.318, 0.270)	0.8739	0.003 (−0.298, 0.303)	0.9845
Preoperative therapy	NAC vs. none	0.360 (0.033, 0.688)	0.0309	0.024 (−0.293, 0.341)	0.8812	0.111 (−0.211, 0.433)	0.4986
	RT vs. none	0.503 (0.171, 0.834)	0.0031	0.457 (0.137, 0.777)	0.0053	0.343 (0.016, 0.670)	0.04
Surgical approach	Robotic vs. laparoscopic	0.162 (−0.077, 0.400)	0.1831	0.094 (−0.139, 0.326)	0.4282	0.243 (0.009, 0.477)	0.0419
Procedure	vLAR vs. LAR	0.437 (0.236, 0.638)	< 0.0001	0.158 (−0.036, 0.352)	0.1106	0.307 (0.108, 0.505)	0.0025
Transanal approach	Ta vs. none	0.860 (0.300, 1.420)	0.0027	0.353 (−0.195, 0.902)	0.2059	0.420 (−0.128, 0.968)	0.1326
Anastomotic leakage	Yes vs. No	0.323 (−0.005, 0.650)	0.0535	0.253 (−0.064, 0.569)	0.117	0.373 (0.050, 0.696)	0.0237
Diverting stoma	Yes vs. No	0.016 (−0.193, 0.224)	0.8823	−0.026 (−0.228, 0.175)	0.7971	0.057 (−0.149, 0.262)	0.5876
Time	6 M vs. preoperative	0.380 (0.262, 0.498)	< 0.0001	0.775 (0.646, 0.904)	< 0.0001	0.511 (0.401, 0.620)	< 0.0001
	12 M vs. preoperative	0.276 (0.157, 0.396)	< 0.0001	0.630 (0.499, 0.761)	< 0.0001	0.459 (0.348, 0.570)	< 0.0001
	24 M vs. preoperative	0.232 (0.099, 0.365)	0.0006	0.458 (0.314, 0.603)	< 0.0001	0.361 (0.238, 0.485)	< 0.0001

*Note: β* coefficients represent adjusted differences in domain‐specific factor scores relative to the reference category, with higher values indicating greater symptom severity in each domain.

Abbreviations: ASA‐PS, American Society of Anesthesiologists physical status; BMI, body mass index; CI, confidence interval; ECOG‐PS, Eastern Cooperative Oncology Group performance status; LAR, low anterior resection; NAC, neoadjuvant chemotherapy; RT, radiotherapy; Ta, transanal approach; vLAR, very low anterior resection.

For Factor 2 (urgency/clustering), radiotherapy was associated with greater severity (*β* = 0.457, 95% CI 0.137–0.777, *p* = 0.005), whereas age ≥ 70 years was associated with lower severity compared with age < 50 years (*β* = −0.257, 95% CI −0.495 to −0.020, *p* = 0.034). No other variables were significantly associated with Factor 2 severity.

Although Factor 3 (gas control) was not included in the primary interpretation of postoperative bowel dysfunction, exploratory mixed‐effects analysis showed that greater severity in this domain was associated with female sex (*β* = 0.200, 95% CI 0.039–0.360, *p* = 0.015), radiotherapy (*β* = 0.343, 95% CI 0.016–0.670, *p* = 0.040), robotic approach (*β* = 0.243, 95% CI 0.009–0.477, *p* = 0.042), very low anterior resection (*β* = 0.307, 95% CI 0.108–0.505, *p* = 0.003), and anastomotic leakage (*β* = 0.373, 95% CI 0.050–0.696, *p* = 0.024).

Postoperative time was a significant determinant of all three domains, with significantly higher symptom severity at 6, 12, and 24 months than at the preoperative assessment.

## 4 Discussion

The present study demonstrated that postoperative bowel dysfunction after sphincter‐preserving surgery is not a uniform entity but comprises multiple symptom domains with distinct correlation patterns and clinical determinants. By integrating item‐level components of the LARS and Wexner scores, we identified three latent domains: an incontinence domain, an urgency/clustering domain, and a gas control domain. Among these, Factors 1 and 2 appeared to represent the principal multidimensional components of postoperative bowel dysfunction. These findings suggest that reliance on composite scores alone may obscure clinically relevant heterogeneity in postoperative bowel symptoms [8].

Previous studies have reported discrepancies between the LARS and Wexner scores [8], but the symptom structure underlying this discordance has remained unclear. By integrating the individual components of both instruments, the present study extends these observations by showing that postoperative bowel dysfunction comprises distinct latent domains and that clinical determinants exhibit different patterns of association across these domains. In particular, vLAR was more strongly associated with the incontinence domain than with the urgency/clustering domain, whereas different age groups showed contrasting associations across these domains. Thus, our findings move beyond the description of score discordance and provide a structural framework that may support domain‐oriented assessment and treatment.

The differential distribution of LARS and Wexner items across the latent domains provides the structural basis for this interpretation. Most Wexner items loaded predominantly on the incontinence domain, whereas LARS items were distributed across the incontinence, urgency/clustering, and gas‐related domains. This pattern highlights a key limitation of interpreting the total LARS score as a single unified measure of postoperative bowel dysfunction. In particular, urgency and clustering contributed strongly to a distinct symptom domain, supporting the view that bowel dysfunction after rectal surgery cannot be fully characterized by a single composite metric [8]. This interpretation is also supported by the component‐level distributions shown in Figure S2.

Our multivariable analysis confirmed that previously reported risk factors for postoperative bowel dysfunction, such as radiotherapy and low anastomotic height, were significantly associated with symptom severity [5, 6, 9, 10]. However, we found that the impact of certain factors differed substantially depending on the symptom domain. For instance, while radiotherapy exacerbated both Factor 1 and Factor 2, surgical factors such as a transanal approach (TaTME) and very low anterior resection (vLAR) significantly worsened Factor 1 (incontinence) but did not exert as large an influence on Factor 2 (urgency/clustering). This difference may be related to the structural effects of an ultra‐low anastomosis and of transanal dissection on the anal sphincter complex. In vLAR, the stapled anastomosis is created within the anal canal itself, which may compromise sphincter function and contribute predominantly to incontinence‐related symptoms.

The most striking domain‐specific difference, however, was observed with patient age. Compared with patients aged < 50 years, those aged 50–70 years had greater severity in the incontinence domain [11], whereas patients aged ≥ 70 years had lower severity in the urgency/clustering domain [7, 12, 13]. This contrasting pattern suggests a difference in the underlying pathophysiology of these domains. The greater incontinence‐domain severity observed in older patients may reflect organic and anatomical vulnerabilities, such as an age‐related decline in pelvic floor reserve. Conversely, the relative prominence of urgency and clustering in younger patients suggests the involvement of functional abnormalities, which may share features with diarrhea‐predominant irritable bowel syndrome (IBS‐D)—such as heightened visceral sensitivity and colonic hypermotility—which generally tend to attenuate with advancing age [14].

Recognizing these distinct symptom domains and their specific risk factors is crucial for establishing personalized treatment strategies. Patients with urgency/clustering‐dominant symptoms (e.g., younger patients) may benefit from pharmacological interventions targeting colonic hypermotility. Recent evidence, including disease‐specific clinical studies, suggests that 5‐HT3 receptor antagonists such as ramosetron may be effective in alleviating urgency and bowel frequency in selected patients with LARS [14, 15, 16]. On the other hand, patients with incontinence‐dominant symptoms may require physical interventions focused on strengthening the pelvic floor and sphincter complex, such as biofeedback or pelvic floor muscle training [17, 18].

Exploratory analysis of Factor 3 suggested that gas‐related symptoms may also represent a partially distinct construct; however, because this domain was narrower in scope and not central to the main clinical question of the study, its interpretation should remain cautious.

This study has several strengths, including its longitudinal design, integration of two widely used symptom instruments at the item level, and the combined use of correlation analysis, exploratory factor analysis, and mixed‐effects modeling. Several limitations should also be acknowledged. First, this was a single‐center study, which may limit generalizability. Second, the three‐factor structure was derived by exploratory factor analysis alone, without confirmatory factor analysis or replication in an independent dataset, and the model fit indices likewise indicated that the solution should be regarded as exploratory; the LARS components were also reweighted for item‐level analysis, so the identified domains should be regarded as hypothesis‐generating and require external validation. Third, the factor analysis pooled observations from 6, 12, and 24 months; longitudinal measurement invariance was not tested, and time‐specific differences in the domain structure therefore cannot be excluded. Fourth, the questionnaire response rate fell to 66.9% at 24 months, and missing responses were accommodated by the mixed‐effects models under a missing‐at‐random assumption without imputation or a formal sensitivity analysis; severity at the later time points may therefore have been underestimated. Fifth, some subgroup estimates, particularly for radiotherapy and transanal procedures, were based on small numbers and should be interpreted cautiously. Sixth, the analysis was restricted to the LARS and Wexner items, which capture neither obstructed defecation nor the consequences of bowel dysfunction that the International Consensus Definition identifies as integral to the LARS construct [1]; measures of obstructed defecation and of quality of life were not available in our database, and the identified domains therefore describe the continence‐ and evacuation‐related aspects of postoperative bowel dysfunction rather than the construct in its entirety. Seventh, although vLAR was defined as an anastomosis created within the anal canal following division of the hiatal ligament, this classification was based on intraoperative assessment by the operating surgeon rather than a standardized, objectively measured anatomical criterion (e.g., distance from the anal verge or the anorectal ring); some inherent ambiguity and limited inter‐surgeon reproducibility in this classification cannot be excluded. Finally, symptom assessment relied on patient‐reported outcomes and may have been influenced by recall bias and subjective perception [19].

In conclusion, postoperative bowel dysfunction after sphincter‐preserving rectal surgery, as captured by the LARS and Wexner instruments, appears to consist of multiple symptom domains with different clinical determinants. Within the continence‐ and evacuation‐related aspects of bowel function, a domain‐based approach may provide a more precise understanding than conventional composite scores and may offer a useful framework for individualized functional assessment and future intervention studies. Future studies should extend this framework to obstructed defecation, the consequences of LARS, and quality of life.

## Author Contributions

**Yoshiko Matsumoto:** writing – original draft, conceptualization, methodology, data curation. **Issei Takeshita:** investigation, validation. **Keiichi Shiokawa:** data curation, investigation. **Kurumi Sahara:** data curation, methodology. **Taro Munechika:** data curation, investigation. **Ken Nagata:** investigation. **Hideki Nagano:** data curation. **Hiroyuki Takahashi:** data curation. **Suguru Hasegawa:** conceptualization, writing – review and editing, supervision.

## Funding

The authors have nothing to report.

## Ethics Statement

The protocol for this research project was approved by the Institutional Review Board of Fukuoka University Hospital (approval number: 16‐5‐04), and the study was conducted in accordance with the Declaration of Helsinki.

## Consent

Informed consent was obtained from all participants.

## Conflicts of Interest

The authors declare no conflicts of interest.

## Supporting information


**Figure S1:** Forest plot of multivariable mixed‐effects analysis for determinants of postoperative bowel dysfunction. The forest plot displays the adjusted beta‐coefficients (dots) and 95% confidence intervals (horizontal lines) for Factor 1 (Incontinence), Factor 2 (Urgency/Clustering), and Factor 3 (Gas control). A beta‐coefficient > 0 indicates greater symptom severity compared with the reference category. For each variable, the category following “vs” denotes the reference group (e.g., “Male” is the reference for “Sex”). Statistical significance is indicated when the 95% confidence interval does not cross the zero line (vertical dashed line). BMI, body mass index; DS, diverting stoma; ECOG‐PS, Eastern Cooperative Oncology Group Performance Status; LAR, low anterior resection; NAC, neoadjuvant chemotherapy; RT, radiotherapy; TaTME, transanal total mesorectal excision; vLAR, very low anterior resection.
**Figure S2:** Longitudinal changes and component analysis of functional scores.
Stacked bar charts showing the chronological changes in (A) Wexner score and (B) LARS score.
(A) Wexner score: The score represents the severity of fecal incontinence, categorized by solid, liquid, and gas incontinence, pad use, and lifestyle alteration. (B) LARS score: The score reflects bowel dysfunction, including gas incontinence, liquid incontinence, bowel frequency, clustering of stools, and urgency. Data are presented as the distribution of each component at four time points: Preoperative (Preop), 6 months (6 M), 12 months (12 M), and 24 months (24 M) postoperatively.

**Table S1:** Reweighting scheme for individual LARS components used in the factor analysis.
**Table S2:** Questionnaire response rates by time point.
**Table S3:** Exploratory factor analysis of Wexner and reweighted LARS items.

## Data Availability

The data that support the findings of this study are available on request from the corresponding author. The data are not publicly available due to privacy or ethical restrictions.
